# Electrodeposited metal-organic framework films as self-assembled hierarchically superstructured supports for stable omniphobic surface coatings

**DOI:** 10.1038/s41598-018-33542-4

**Published:** 2018-10-18

**Authors:** Jakob Sablowski, Julia Linnemann, Simone Hempel, Volker Hoffmann, Simon Unz, Michael Beckmann, Lars Giebeler

**Affiliations:** 1 Chair of Energy Process Engineering, Institute of Process Engineering and Environmental Technology, Technische Universität Dresden, 01062 Dresden, Germany; 2 Leibniz Institute for Solid State and Materials Research (IFW) Dresden e.V., Institute for Complex Materials, Helmholtzstraße 20, 01069 Dresden, Germany; 3 Chair of Physical Chemistry, Technische Universität Dresden, Bergstraße 66b, 01069 Dresden, Germany; 4 Institute of Construction Materials, Technische Universität Dresden, Georg-Schumann-Straße 7, 01187 Dresden, Germany

## Abstract

Superhierarchically rough films are rapidly synthesised on metal substrates via electrochemically triggered self-assembly of meso/macroporous-structured metal-organic framework (MOF) crystals. These coatings are applied to immobilise a functional oil with low surface energy to provide stable coatings repellent to a wide range of hydrophobic as well as hydrophilic fluids. Such omniphobic surfaces are highly interesting for several applications such as anti-fouling, anti-icing, and dropwise condensation, and become easily scalable with the presented bottom-up fabrication approach. As investigated by environmental scanning electron microscopy (ESEM), the presented perfluorinated oil-infused Cu-BTC coating constitutes of a flat liquid-covered surface with protruding edges of octahedral superstructured MOF crystals. Water and non-polar diiodomethane droplets form considerably high contact angles and even low-surface-tension fluids, e.g. acetone, form droplets on the infused coating. The repellent properties towards the test fluids do not change upon extended water spraying in contrast to oil-infused porous copper oxide or native copper surfaces. It is discussed in detail, how the presented electrodeposited MOF films grow and provide a proficient surface morphology to stabilise the functional oil film due to hemiwicking.

## Introduction

Functional surfaces with designable wetting properties enable varying degrees of liquid repellency towards water, oils or even low-surface-tension fluids. The surface tension of a liquid describes the change of the surface free energy upon changing the surface area (for constant temperature and volume)^1^. This refers to the characteristic behaviour of liquids, like water, to form spherical droplets to minimise the surface area and attain an energetically favourable state in which as many molecules of the liquid as possible are surrounded by equal molecules “inside” the volume of the droplet. In this way, intermolecular interactions, such as hydrogen bonding in the case of water, determine the surface tension of a liquid. For droplets on ideally flat, smooth, and homogenous solid surfaces, the Young contact angle *θ*_*Y*_ between the solid surface (liquid/solid interface) and the surface of the droplet (liquid/gaseous interface) depends on (a) the surface tension of the liquid holding the droplet together, (b) the free surface energy of the solid phase pushing the droplet apart towards the solid phase, and (c) the interfacial tension between the solid and the liquid phase^2^. The interfacial tension *γ*_*sl*_ then again results from the surface tension of the solid *γ*_*s*_, the surface tension of the liquid *γ*_*l*_, and interactions of similar type between the phases. These interfacial interactions can be described by the geometric mean of polar (${\gamma }_{s}^{p}$, ${\gamma }_{l}^{p}$) and dispersive (non-polar, ${\gamma }_{s}^{d}$, ${\gamma }_{l}^{d}$) contributions, relating to the concept of hydrophilic and hydrophobic interactions^3^:

1 $${\gamma }_{sl}={\gamma }_{s}+{\gamma }_{l}-2(\sqrt{{\gamma }_{s}^{p}\cdot {\gamma }_{l}^{p}}+\sqrt{{\gamma }_{s}^{d}\cdot {\gamma }_{l}^{d}})$$

Hence, it is possible to obtain stable droplets of both hydrophilic (e.g. water) and hydrophobic liquids (e.g. diiodomethane) and even liquids with low surface tension (e.g. acetone), if the free surface energy of the solid surface is sufficiently low. Furthermore, the wetting properties of surfaces are influenced by the surface morphology as roughness and air pockets in between features of the solid surface and the liquid phase affect the contact angle.

In brief, so-called omniphobic surfaces are non-wetting towards a wide range of fluids. Therefore, these surfaces have gained interest from fields such as energy and heat transfer engineering, medical devices, transportation and construction^4–6^.

**Figure 1:**
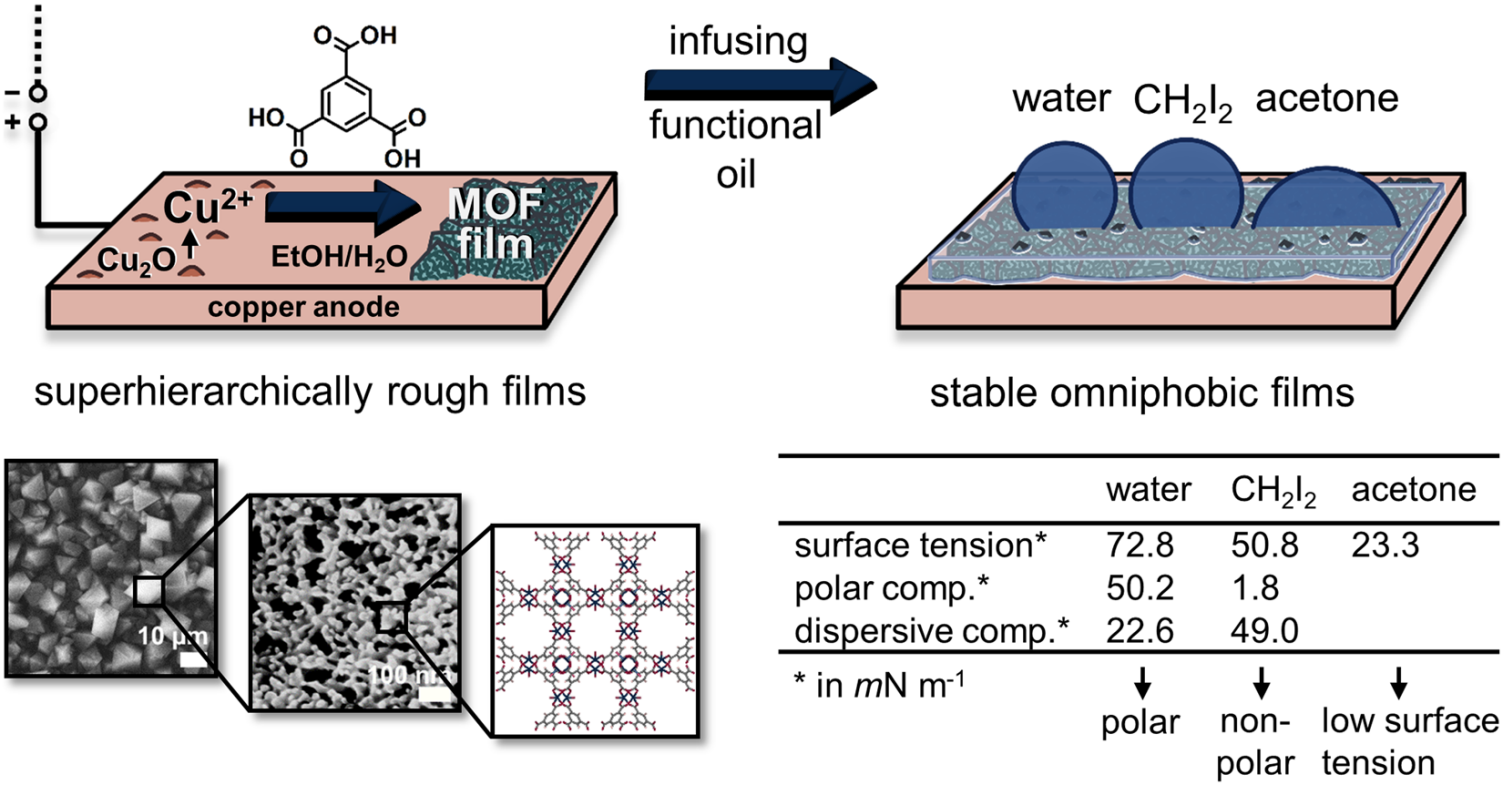
Electrodeposition of rough MOF films as support matrix for stable omniphobic oil-infused films (overview scheme). Cu-BTC framework films exhibiting roughness and porosity with morphological features in several length scales are coated electrochemically on Cu sheets providing for stable trapping of a functional oil. Without any additional modification, oil-infused coatings are obtained repelling polar (water) and non-polar (CH_2_I_2_) test fluids as well as solvents with low surface tension (acetone) even after comprehensive water spraying. Values for surface tension, polar component and dispersive component of water and CH_2_I_2_ at 20 °C according to ref.^69^ and surface tension of acetone at 25 °C according to ref.^70^.

As displayed in Fig. 1, in this study, copper sheets were electrochemically coated with superhierarchical metal-organic framework (MOF) films and infused with a perfluorinated oil obtaining stable surfaces with omniphobic properties. Such oil- or lubricant-infused surfaces are bio-inspired materials which resemble the surface of the *Nepenthes* pitcher plant^7^ and have been shown to repel various liquids^8,9^. Potential applications of oil-infused surfaces include anti-fouling^10–13^, anti-icing^14–16^, drag reduction^17^ and self-cleaning^8,9,18^ surfaces as well as heat transfer enhancement *via* dropwise condensation^19–22^. In contrast to other omniphobic surfaces which rely on re-entrant surface morphologies^23–25^, oil-infused surfaces maintain their liquid-repellent properties under pressure^8^ and during condensation^19,21^.

In principle, an oil exhibiting low surface tension is infused into a chemically modified rough surface structure to immobilise the oil, thus, functionalising the surface with the oil’s properties. The additional chemical treatment prior to oil infusion commonly introduces hydro- or omniphobic organic long-chain molecules to the surface by silanisation^8,19–21,26–28^. Thereby, the interfacial tension of the oil and the solid support is lowered by increased chemical affinity^27^.

Using a metal-organic framework (MOF) material as solid phase to trap a functional oil exploits several favourable properties of this material class. MOFs are porous crystalline compounds which consist of metal ions or metal clusters linked by organic molecules which form coordinative bonds to the metal centres with their functional groups. In comparison to metal or ceramic materials, which are characterised by very strong types of binding (e.g. covalent, ionic, or metallic), MOFs exhibit moderate free surface energies and their wetting properties can be greatly determined by the organic ligand molecules due to their spatial extent. Hydrophobic and superhydrophobic linkers were employed to synthesise moisture-resistant MOF powders for adsorption or separation applications^29–31^. Furthermore, amine^32^- and vinyl^33^-containing MOFs were postsynthetically modified with hydrophobic alkyl chains or omniphobic perfluoroalkyl chains by chemical reactions. Recently, rationally designed linker molecules have even been used to obtain MOF crystals with superhydrophobic corrugated surfaces due to periodic arrangement of hydrocarbon moieties of the linker molecules at MOF crystal surfaces^34,35^.

However, the synthetic effort to obtain such linker molecules and MOFs is high and further processing technologies would be required to fabricate coatings which can be infused with an omniphobic oil. Therefore, we aimed to use the well-known Cu-1,3,5-benzene-tricarboxylate (BTC) framework (HKUST-1)^36^ and synthesise this compound in the form of superstructured crystals^37,38^ with hierarchical porosity across the nanoscale [referring to macro- (>50 nm), meso- (2–50 nm) and micropores (<2 nm) as defined by the IUPAC], which may be able to sufficiently stabilise an omniphobic oil film due to their morphology despite their rather hydrophilic nature (see discussion part for detailed explanation).

Researchers at BASF developed an electrochemical synthesis method for MOF powders, in which metal ions are provided to a linker molecule containing electrolyte solution by applying an anodic potential to a metal electrode^39^. Further studies achieved the electrodeposition of MOF films at the anodised electrode, thereby providing a mild and scalable approach to produce MOF coatings with short growth times^40,41^ employable for sensor^40,42^, (micro)electronical^43,44^, and energy storage^45,46^ applications.

Herein, we present superhierarchical Cu-BTC films electrochemically grown on copper surfaces in 10 min. While the shape of the octahedral crystals forming the MOF coating provides roughness in the micrometer scale, the hierarchical porosity of these superstructured crystals originates from additional meso/macro voids and micropores of the crystalline framework. Such crystal morphologies may potentially increase the performance of MOF films in certain applications due to enhanced mass transport in crystals while sustaining the properties associated with the nanoscaled micropores of a crystalline MOF material. In this study, superhierarchical Cu-BTC coatings are used to immobilise a perfluorinated oil with low surface tension to obtain omniphobic surfaces. No additional chemical functionalisation was applied to the MOF-coated surface prior to oil infusion. As investigated by contact angle measurements and spray testing, the resulting functionalised surfaces repel polar and non-polar liquids while stably trapping the oil. This was not the case for further examined native copper surfaces and roughened anodised copper oxide surfaces. The wetting experiments and environmental scanning electron microscopy (ESEM) conducted to characterise the appearance of the oil-infused MOF film suggest the superhierarchical morphological features in several size scales as key aspect of the coating design.

The combination of omniphobic wetting properties, stable trapping of a perfluorinated oil and a fabrication method, which can be applied to metallic and other conductive surfaces on large, geometrically complex components like heat exchangers, is a promising starting point for cost-effective industrial applications in the field of anti-fouling, anti-icing and dropwise condensation.

## Results

### Electrodeposition of hierarchically superstructured Cu-BTC framework coatings

Films of the Cu-BTC framework (HKUST-1) were grown on pure copper foil by potentiostatically applying an anodic potential to the foil for 10 min at 50 °C in an electrolyte solution containing 1,3,5-benzene-tricarboxylic acid (H_3_BTC) as linker molecule precursor. As an additional additive, methyl-tributyl-ammonium methyl-sulphate (MTBS) comprises a quaternary ammonium cation with surfactant properties and increases the conductivity of the electrolyte. Note that the used solvent mixture of ethanol and water (v/v 3:1) also contains dissolved oxygen as well as water, which act as oxidising and O-providing agents.

**Figure 2:**
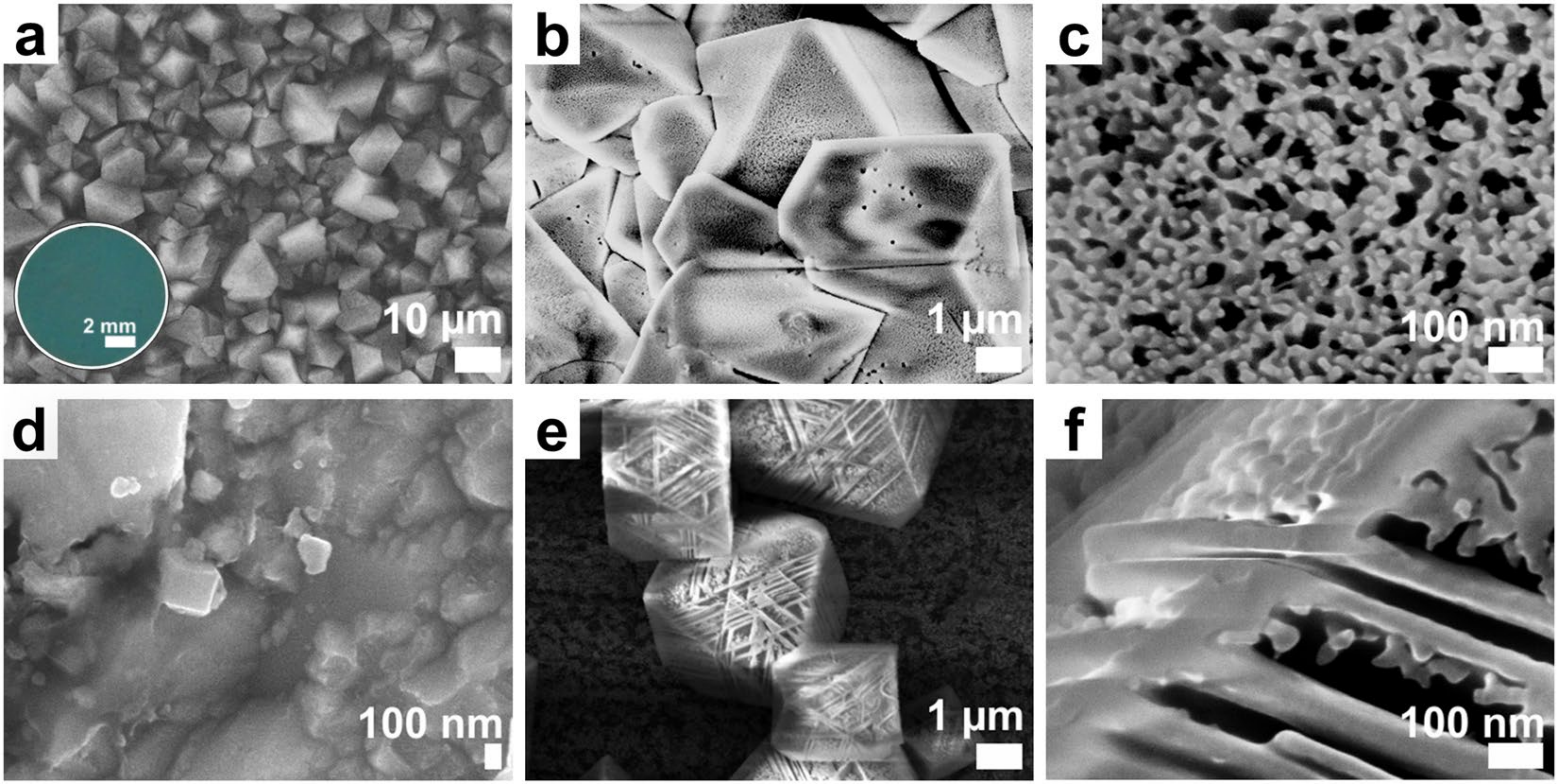
Micro- and nanostructure of electrodeposited MOF films. (**a**–**c**) SEM images of electrodeposited Cu-BTC framework films showing (**a**,**b**) the top view on intergrown octahedral crystals, (**c**) a crystal surface at high magnification and (inset in **a**) a photograph of the MOF film. (**d**) SEM image of a Cu foil anodised in an electrolyte not containing linker molecules and (**e**,**f**) SEM images of other Cu-BTC framework films electrodeposited at room temperature showing (**e**) the top view on octahedral crystals and (**f**) a crystal edge at high magnification. The scale bars are (**a**) 10 µm, (**b**,**e**) 1 µm and (**c**,**d**,**f**) 100 nm.

**Figure 3:**
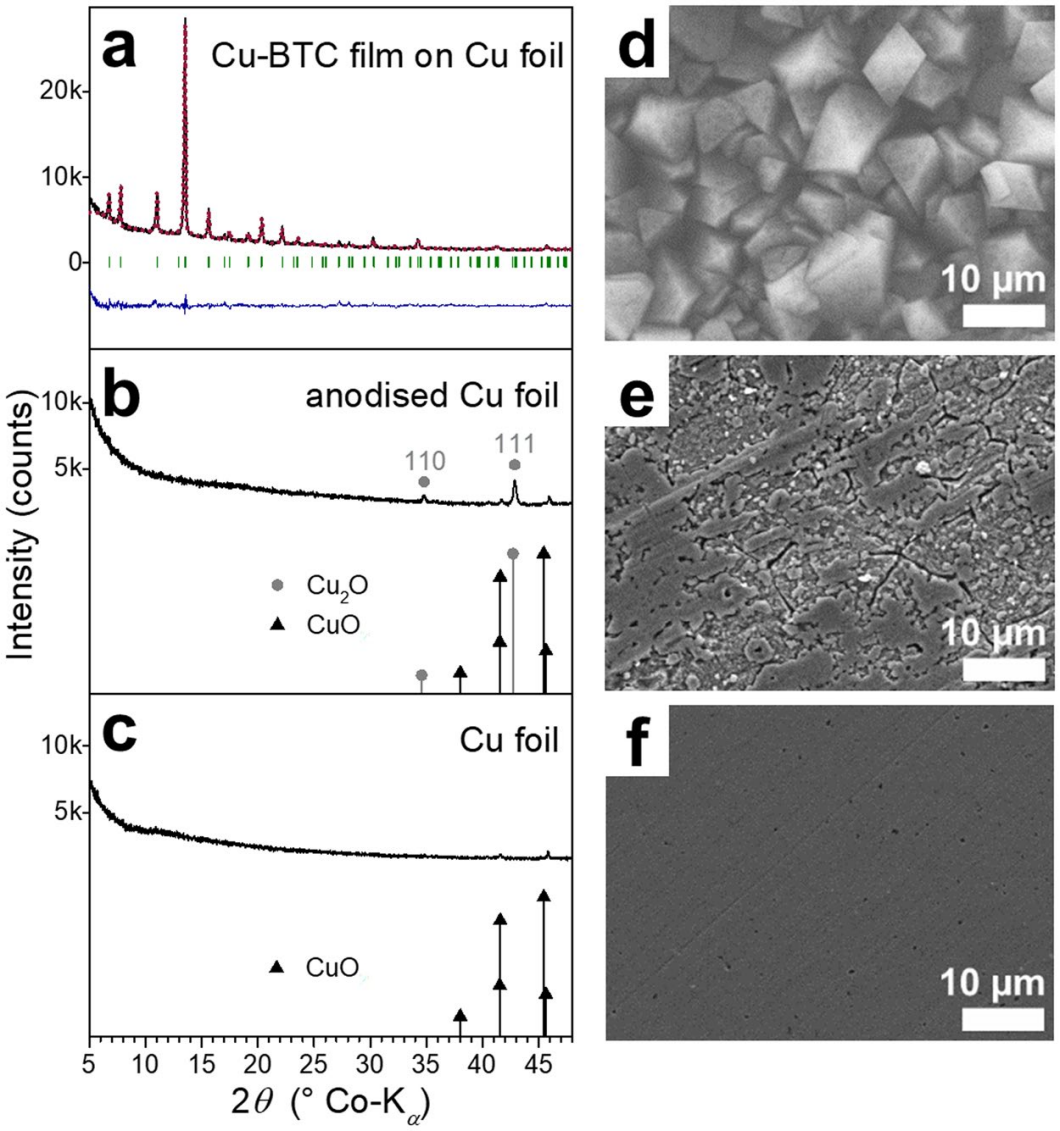
Comparison of the surfaces of electrochemically modified and native Cu foils. (**a**–**c**) XRD patterns and (**d**–**f**) SEM images of (**a**,**d**) an electrodeposited Cu-BTC framework film on Cu foil and results of the structure analysis (using the structure model of HKUST-1^36^, red: calculated pattern, green: Bragg positions, blue: difference plot), (**b**,**e**) a Cu foil anodised in an electrolyte not containing linker molecules and reference patterns (Cu_2_O *Pn*-3*m*^71^, CuO *C*2/*c*^72^) and (**c**,**f**) a native Cu foil and reference patterns (CuO as in **b**). The scale bars are (**d**–**f**) 10 µm.

By this simple electrochemical treatment, a coating with a highly porous superhierarchical morphology is obtained providing for stable immobilisation of a functional oil. As shown in Fig. 2a, electrodeposited films homogenously cover the Cu foil and consist of octahedral, differently aligned crystals in the size range of several micrometres. The crystals are intergrown and lead to microroughness of the MOF coating. By optical profilometry, the average surface roughness (*R*_*a*_) of the Cu-BTC coating was determined as 2.23 ± 0.24 μm, being significantly higher compared to a native Cu foil (Supplementary Table 1 and Fig. 1). Typical islands’ heights are around 5 μm with similar lengths, being in accordance with the scanning electron microscopy (SEM) image. SEM with higher magnification (Fig. 2b,c) reveals that the Cu-BTC crystals feature a distinct morphology with additional macro- and mesopores. More specifically, they are hierarchically superstructured crystals made up of interconnected nanosized MOF entities. These MOF parts are crystalline. The respective X-ray diffraction (XRD) pattern of the coating (Fig. 3a) displays sharp signals and is assigned to the Cu-BTC framework (HKUST-1) as shown by structure analysis according to the Rietveld method with the corresponding structure model^36^. The characteristic geometrical shape and the XRD results make it evident that the Cu-BTC material is deposited in the form of microcrystals assembled from smaller nanoentities while maintaining a higher-order superstructure; but not as disordered aggregates of MOF nanoparticles. At room temperature, the electrodeposition process yields coatings in which the Cu-BTC crystals are much less intergrown (Supplementary Fig. 2a). As Fig. 2e,f demonstrate, the nanomorphology (macro- and mesopores) is similar to the crystals deposited at 50 °C but displays less regularity and several nanometre thick solid “walls”.

An electrochemical surface treatment without linker molecules present in the electrolyte was subjected to copper foils to further investigate the effect of the anodic treatment on the foil gaining insights into the coating process. Moreover, by this treatment, another type of roughened surfaces on Cu foil is produced, which was also infused by the perfluorinated oil and characterised regarding the omniphobic properties. Similarly to the Cu-BTC coating process, the Cu foil was potentiostatically anodised for 10 min at 50 °C in an ethanol/water (v/v 3:1) solution containing the quaternary ammonium salt MTBS but not H_3_BTC. Thereby, compact morphological features in the nanoscale, besides larger ones, are introduced to the foil surface (Fig. 2d), which may function as precursor particle or rather preferred nucleation site in case of the linker-containing electrolyte. In Fig. 3e, a SE micrograph of a larger area is shown in comparison to the untreated, native Cu foil (Fig. 3f) and a Cu-BTC coating (Fig. 3d, Fig. 2a-c). After 10 min of anodisation without H_3_BTC, cracks are formed on the foil surface. Some patches still resemble the native foil with some additional voids, whereas other patches are electrochemically diluted to a much higher extent.

The XRD pattern of native Cu foil (Fig. 3c) exhibits very weak signals assigned to cupric oxide (CuO) as copper forms a very thin passivation layer in atmospheric conditions. After the anodisation with just MTBS-containing electrolyte, additional signals of cuprous oxide (Cu_2_O) appear in the XRD pattern (Fig. 3b). Remarkably, Cu_2_O signals are not observed for the Cu-BTC coating process in which H_3_BTC is added to the electrolyte indicating the consumption of Cu_2_O by the MOF formation. These findings were furtherly confirmed by Raman spectroscopy measurements (Supplementary Fig. 3) in which several bands assigned to Cu_2_O for the anodised foil and a band at ca. 640 cm^−1^ assigned to CuO for the native and the anodised foils^47–50^ were observed. Thus, anodising a copper foil under similar conditions but with linker-free electrolyte introduces a rough Cu_2_O/Cu surface but does not provide surfaces with such a hierarchical nanoroughness as observed for the Cu-BTC framework coatings.

### Omniphobic wetting properties and stability of oil-infused support surfaces

**Figure 4:**
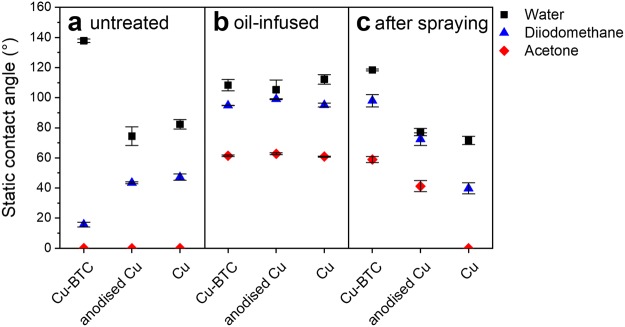
Omniphobic properties and stability of oil-infused MOF films compared to electrochemically modified and native Cu foils. Measured static contact angles of water, diiodomethane and actone on (**a**) untreated and (**b**–**c**) oil-infused samples (**b**) before and (**c**) after 30 min spray testing. Data points on top of the x-axis indicate complete wetting of the surface. Error bars represent the combined standard uncertainty^73^.

Without any additional surface functionalisation, an as-deposited Cu-BTC coating exhibits a water contact angle of 137.8° ± 1.2° and a diiodomethane contact angle of 15.7° ± 1.6°. These contact angles result from the high surface roughness of the electrodeposited Cu-BTC films. Roughness leads to an increased contact angle, if the base material exhibits a Young contact angle *θ*_*Y*_ > 90° and to a decreased contact angle, if the base material exhibits *θ*_*Y*_ > 90° either due to a Wenzel state^51^ or a Cassie state^52^. In both possible cases, the surface roughness of the electrodeposited Cu-BTC films enhances the hydrophobic and oleophilic properties of the base material effectively increasing the water contact angle and decreasing the diiodomethane contact angle compared to the Young contact angle of the same material. The test fluid with low surface tension, acetone, completely wets the Cu-BTC film, as well as the native copper foil and the anodised copper foil (Fig. 4a).

After the application of the perfluorinated oil to each surface, all three surfaces exhibit similar omniphobic wetting properties (Fig. 4b). Thus, the wetting properties of the infused surfaces are mainly governed by the perfluorinated oil. This observation identifies the underlying surface as not property-determining, if it is completely covered by such a functional oil.

The similarity of the surfaces’ wetting behaviour is most likely based on the high viscosity of the applied Krytox oil (522 mm^2^ s^−1^ at 20 °C)^53^. Since the contact angles were measured within the first 2 seconds after the droplet placement, the droplets have not yet displaced the highly viscous oil film. However, about 25 seconds after the drop placement, a slight sinking motion of the droplet was observed on all three surfaces (Supplementary Figs 4–6). To further investigate the displacement of the functional oil, spray tests were conducted with all oil-infused surfaces. After being submitted to a water spray for 30 minutes, the contact angles on the native copper foil and the anodised copper foil are significantly decreased indicating the expected displacement of the oil film (Fig. 4c). In contrast, the omniphobic wetting behaviour of the infused Cu-BTC film was retained even after the spray test.

**Figure 5:**
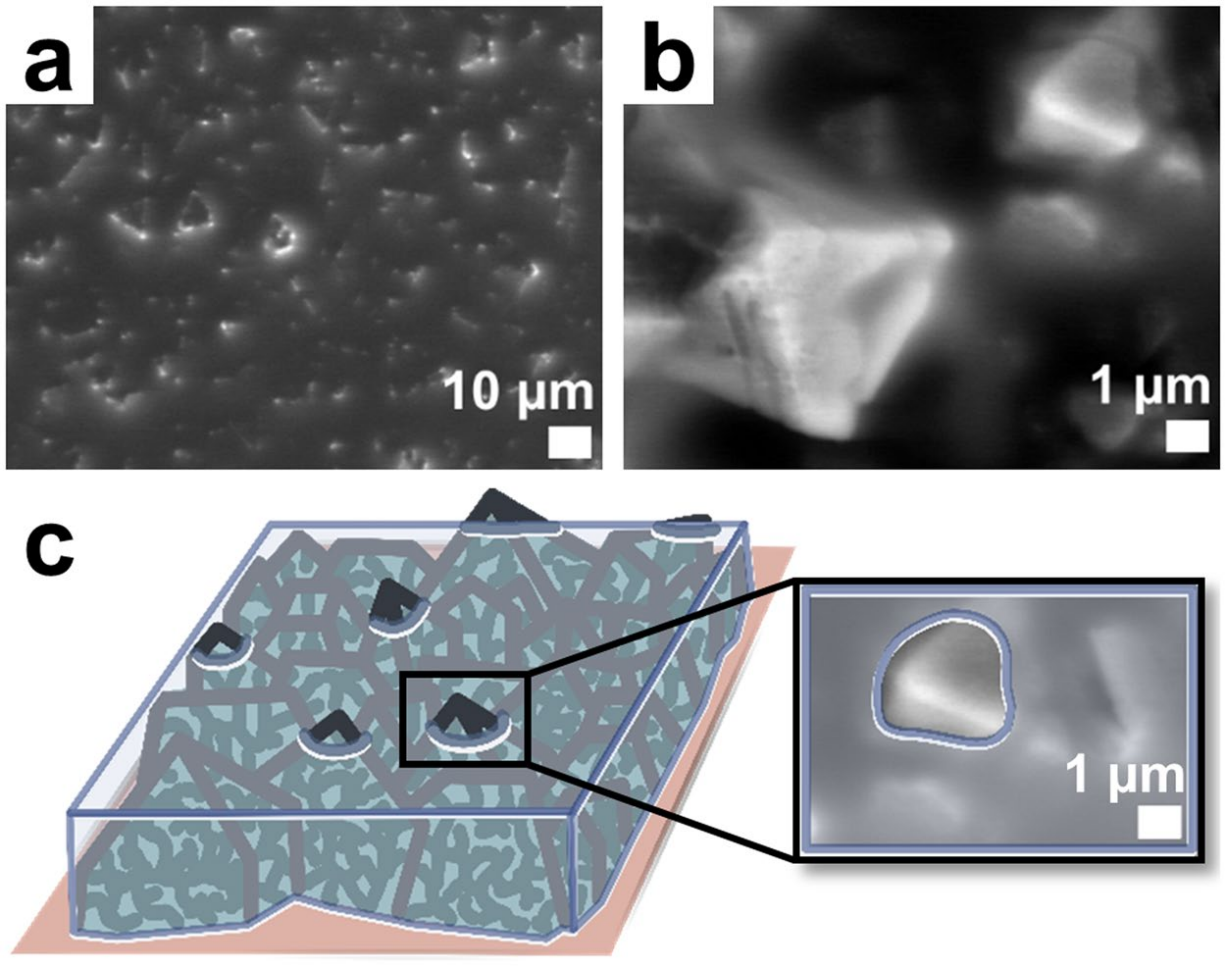
Appearance of omniphobic oil-infused MOF films. (**a**,**b**) ESEM images of electrodeposited Cu-BTC framework films infused with perfluorinated oil and (**c**) scheme and coloured ESEM image section to illustrate how the perfluorinated oil is localised within and on the Cu-BTC framework films. The scale bars are (**a**) 10 µm and (**b**) 1 µm.

The observed sustainment of the omniphobic properties indicates that only marginal or even no drainage of the functional oil from the superstructured Cu-BTC surfaces occurs. The thereby-suggested stability of the oil film at the MOF coating is furtherly confirmed by ESEM measurements taken after the spray tests. As shown on the micrographs in Fig. 5, the sample surface is still covered by an oil film while some of the bigger MOF crystals protrude above the top. The parts of the MOF crystals enclosed by the oil film are also imaged as the electron beam shines through the oil and respective secondary electrons are detected. However, the meniscus of the oil is recognised around the protruding crystal edges in Fig. 5b upon the brighter and sharper appearance of these edges. It is assumed that the nanoscaled meso- and macro voids of the Cu-BTC crystal surfaces are also filled with oil. At first, the nanomorphology is not observed. After extended irradiation with the electron beam, the voids become visible again, suggesting that the oil vanishes by this treatment.

To further characterise the wetting and liquid adhesion, dynamic contact angles were measured on an infused Cu-BTC film (Table 1). For the unpolar test liquid diiodomethane, the coating exhibits a low contact angle hysteresis of 7.0° ± 0.5°. Thus, a “slippery” wetting behaviour with high droplet mobility is observed. This property is characteristic for superomniphobicity referring to omniphobic surfaces with very high contact angles and low contact angle hystereses of *Δθ* < 10° (similar to the concept of superhydrophobicity)^21,54,55^. However, the rather high contact angle hystereses of 101.4° ± 3.0° for water and 44.1° ± 1.1° for acetone indicate that these liquids do not easily roll off the surface.

**Table 1:** Dynamic contact angles on oil-infused MOF films.

Liquid	*θ*_a_ (°)	*θ*_r_ (°)	*Δθ* (°)
Water	107.1 ± 1.3	5.7 ± 2.7	101.4 ± 3.0
Diiodomethane	99.21 ± 0.4	92.2 ± 0.4	7.0 ± 0.5
Acetone	65.7 ± 0.4	21.7 ± 1.0	44.1 ± 1.1

Advancing contact angle, *θ*_a_, receding contact angle, *θ*_r_, and contact angle hysteresis, *Δθ*, of water, diiodomethane and acetone on oil-infused Cu-BTC framework films.

The oil film/MOF surface exhibits omniphobicity at a high stability without any additional functionalisation of the Cu-BTC support coating prior to the infusion of the perfluorinated oil. Instead, trapping of the oil may be ensured by the distinct porous morphology of the Cu-BTC framework coating as discussed in the following section.

## Discussion

### Stability of the oil-infused surfaces and the porosity of support films

The properties of the oil-infused surface with regard to liquid drainage and film stability are closely related to the thermodynamic state of a single droplet which is placed on the infused surface.

**Figure 6:**
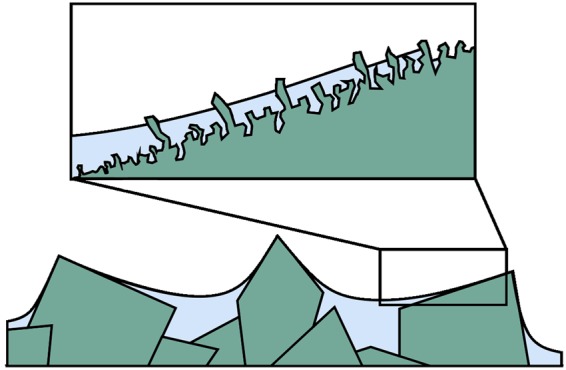
Lubricant infusion of the MOF films through hemiwicking. Scheme to illustrate the propagation of the perfluorinated oil on the superhierarchically structured MOF crystals. Surface features are not to scale.

States in which the perfluorinated oil completely encapsulates the rough surface of the MOF are only stable, if the oil completely wets the MOF^26^. This condition would be met, if the Young contact angle between the oil and an ideal flat surface with the same surface free energy as the MOF equalled zero in the presence of air, i. e. *θ*_*OS*(*a*)__,__*Y*_ = 0°. Due to the moderate surface free energy of the Cu-BTC framework material, which was not subjected to further modification or treatments to achieve a low surface free energy, we can assume *θ*_*OS*(*a*)__,__*Y*_ > 0°. Therefore, the omniphobic oil does not encapsulate the surface completely but rather propagates inside the texture leaving some of the MOF superstructured crystals obtruded as shown in Fig. 6.

As deduced by Bico *et al*.^56^, this phenomenon of *hemiwicking* occurs, if the contact angle of the oil *θ*_*OS*(*a*)__,__*Y*_ is smaller than a critical contact angle *θ*_*c*_.

2 $${\theta }_{OS(a),Y} < {\theta }_{c}$$

with

3 $$\cos \,{\theta }_{c}=\frac{1-{\varphi }_{S}}{r-{\varphi }_{S}}$$

In Equation (3), *ϕ*_*S*_ denotes the fraction of the projected surface area which is not covered by the oil and *r* denotes the ratio of the total surface area to the projected surface area. For superhierarchical surface structures like the Cu-BTC films, *ϕ*_*S*_ is not accessible for measurements but, by definition, takes on values between 0 and 1 resulting in

4 $$\cos \,{\theta }_{c} < \frac{1}{r}$$

and therefore

5 $$\cos \,{\theta }_{OS(a),Y} > \frac{1}{r}$$

as a sufficient condition for hemiwicking^9^. For highly porous surfaces with roughness ratios *r* → ∞, such as the electrodeposited superhierarchical Cu-BTC film, the critical contact angle approaches π/2.

In order to stably trap the omniphobic oil in the surface texture, not only in the presence of air but also in the presence of a second liquid, e.g. if a drop of a second liquid *b* is placed on the oil-infused surface, the condition

6 $${\theta }_{OS(b),Y} < {\theta }_{c}$$

must be fulfilled. As discussed above, ${\theta }_{c}=\frac{\pi }{2}$is assumed for the highly porous MOF surface morphologies and therefore ${\theta }_{OS(b),Y} < \frac{\pi }{2}$. This condition is fulfilled, if the liquid *b* has a lower tendency to wet the surface than the oil used for the infusion, i.e. *θ*_*bS*(*a*)__,__*Y*_ > *θ*_*OS*(*a*)__,__*Y*_.

Therefore, if a surface is extremely porous (resulting in high values of *r*), an oil-infused film is stable in the presence of all immiscible liquids which wet the surface less than the oil used for infusion. Thus, stable omniphobic properties are achieved in certain cases without any additional functionalisation of a rough surface to increase the chemical affinity between the functional low-surface-tension fluid and the solid surface. Due to their highly porous, superhierarchical surface morphology, the electrodeposited Cu-BTC films provide an excellent support for the oil film resulting in the superior stability presented in Fig. 4c.

### Electrochemically triggered formation of hierarchically superstructured Cu-BTC crystals

As explained above, the additional macro- and mesopores of the superstructured Cu-BTC crystals are assumed to be a crucial feature regarding the application of the coatings for oil-infused omniphobic films. In this study, Cu-BTC coatings are synthesised by an electrodeposition approach. Due to the observed Cu_2_O formation in the linker-free electrolyte (Fig. 3b, Supplementary Fig. 3a), it is concluded that Cu^2+^ ions are not only provided to the electrolyte solution *via* reaction (7). The anodic potential applied to the Cu foil triggers also the oxidation process (8) in which the Cu(I) oxide species is formed. Cu_2_O may be furtherly oxidised forming Cu^2+^ ions (9) as the potential range of stability of Cu_2_O is rather narrow^57^.

7 $${\rm{Cu}}\to {{\rm{Cu}}}^{2+}+2{{\rm{e}}}^{-}$$

8 $$2{\rm{C}}{\rm{u}}+{{\rm{H}}}_{2}{\rm{O}}\to {{\rm{C}}{\rm{u}}}_{2}{\rm{O}}+2{{\rm{H}}}^{+}+2{{\rm{e}}}^{-}$$

9 $${{\rm{Cu}}}_{2}{\rm{O}}+2{{\rm{H}}}^{+}\to 2{{\rm{Cu}}}^{2+}+{{\rm{H}}}_{2}{\rm{O}}+2{{\rm{e}}}^{-}$$

If H_3_BTC is added to the electrolyte solution, reaction (9) is promoted because of the lower pH value and the consumption of Cu^2+^ due to Cu-BTC framework formation. Therefore, signals of cuprous oxide (Cu_2_O) do not appear in the XRD pattern after the MOF-coating treatment with the H_3_BTC-containing electrolyte (Fig. 3a). Cu_2_O is also known to disproportionate to Cu and Cu^2+^ in the presence of organic acids^57^. As there is dissolved oxygen present in the electrolyte, Cu_2_O may also be chemically oxidised to form Cu-BTC *via* reaction (10).

10 $$6{{\rm{C}}{\rm{u}}}_{2}{\rm{O}}+8{{\rm{H}}}_{3}{\rm{B}}{\rm{T}}{\rm{C}}+3{{\rm{O}}}_{2}\to 4{{\rm{C}}{\rm{u}}}_{3}{({\rm{B}}{\rm{T}}{\rm{C}})}_{2}\cdot 3{{\rm{H}}}_{2}{\rm{O}}$$

In line with these conclusions, Schäfer *et al*.^58^ recently found Cu-BTC formation to occur only, if a Cu_2_O layer is present on the copper anode or, if the electrolyte contains O_2_ and/or H_2_O to enable intermediate Cu_2_O formation. Furthermore, Cu_2_O was detected along with anodically deposited Cu-BTC on Cu foil^59^.

To achieve crystalline powder of MOF-5^60^ (Zn_4_O(BDC)_3_, BDC = 1,4-benzene-dicarboxylate) with additional heterogeneously arranged macro- and mesopores, a certain amount of a carboxylate-terminated structuring agent (4-(dodecyloxy)benzoic acid (DBA)) was added to the synthesis solution in order to locally hamper crystal growth without making use of templating mesophases^61^. Detailed investigations on the formation mechanism revealed an intermediate growth stage of cubic crystals with a textured surface appearing similarly to the crystals we obtained at room temperature comprising solid “nanowalls” (Fig. 2e,f). Greer *et al*.^62^ concluded that the crystallisation proceeds *via* a reversed crystal growth route in which oriented aggregation of initially formed nanocrystallites is promoted by DBA also acting as surface ligand and spacer. In later stages, recrystallization occurs from the surface to the core.

To chemically synthesise crystalline macro- and mesostructured Cu-BTC 3D-networks, Cu_2_O nanocubes were employed as spatially inhomogenous metal ion source in combination with the non-ionic polymer poly(vinylpyrrolidone) (PVP) as a necessary structure-directing agent^63^. In this regard, morphological nanofeatures on the Cu foil or detaching nanoparticles, as seen in Fig. 2d, may play a role especially for the nucleation of the electrochemically triggered formation of superstructured Cu-BTC crystals. The quaternary ammonium ions of the MTBS salt assumingly influence the crystal growth either through local inhibition of the crystal growth or through space blocking. The nanostructuring effect of quaternary ammonium surfactants is well known in the electrochemical synthesis of metal or metal oxide films^64,65^. The possible anodic formation of oxygen bubbles by water decomposition at the electrode surface may also affect the coating morphology considering such bubbles to take up space and promote the chemical conversion of Cu_2_O to Cu-BTC.

Generally, in homogenous synthesis, Cu-BTC framework crystallisation happens without an induction period independently of the chosen temperature and nucleation proceeds continuously, dominating the crystallisation rate under solvothermal^66^ as well as open conditions^67^. Accordingly, for the electrodeposited films, different crystallite sizes were observed. The particular interplay of nucleation and growth rate, which are influenced by electrochemical parameters and temperature, may be crucial for the electrochemically triggered formation of superstructured Cu-BTC crystals, likely depending on nanoparticulate Cu_2_O precursors, self-assembly of nanocrystallites, and spatially confined crystal growth inhibition. Therefore, a deeper understanding of the electrochemically triggered crystallisation of MOF films represents a highly interesting subject for future studies.

## Conclusion

Due to their superhierachical porous morphology, the presented electrodeposited Cu-BTC framework coatings constitute an excellent surface structure for the trapping of a functional oil film. The highly porous surface structure of the MOF crystals in combination with an appropriately viscous oil causes superior stability of the oil film, even if the oil-infused surface is wetted by another immiscible fluid. Utilising the spreading of the oil on the porous MOF crystal surfaces driven by capillary forces (hemiwicking), anodically deposited MOF supports on copper foil were stably infused with a perfluorinated oil exhibiting low surface tension.

The resulting surface repels polar and non-polar liquids with various surface tensions and is not altered by extended water spraying. The formation mechanism of the hierarchically superstructured crystal film during the electrochemical coating procedure is likely based on the suitable relation of nucleation and growth rate, intermediate cuprous oxide (Cu_2_O), the nanocrystalline nature in early stages of the electrodeposition process, and surfactant electrolyte additives. Omniphobic properties and stability were achieved without any additional modification of electrodeposited MOF films, enabling a scalable one-step fabrication of such support coatings on many metal surfaces even with complex geometries. By further research, the chemical and topographical properties of MOF support films can be adjusted for specific applications.

## Methods

### Materials

Copper foils with a thickness of 0.15 mm were produced in-house by rolling and annealing of electrolytic copper. 1,3,5-benzene-tricarboxylic acid (H_3_BTC, 95%, Sigma-Aldrich), methyl-tributyl-ammonium methyl-sulphate (MTBS, ≥95%, Sigma-Aldrich), ethanol (absolute, analytical grade, Merck) and the perfluorinated oil Krytox GPL 105 (DuPont) were used as received without further purification. Deionised ultrapure water (κ < 0.06 µS cm^−1^) was used for electrolytes and cleaning procedures. Contact angle measurements were performed with deionised water (κ < 1.0 µS cm^−1^), diiodomethane (CH_2_I_2,_ 99%, Alfa Aesar) and acetone (technical grade, >98%, BHD Prolabo) as testing fluids.

### Preparing omniphobic surfaces

Copper foils were cleaned by ultrasonication in soapy water for 10 min, rinsing with deionised water and ethanol, followed by ultrasonication in ethanol for 10 min and drying in air with a blow-dryer.

The Cu foil surfaces were electrochemically modified with a Heka PG310 potentiostat in a three electrode setup using a platinum counter electrode and a mercury sulphate reference electrode (MSE) which was connected to the electrochemical cell by a salt bridge (0.1 M Na_2_SO_4_, agar). Hierarchically structured Cu-BTC framework coatings were electrodeposited potentiostatically on Cu foil applying a potential of 0.5 V *vs*. MSE for 10 min at 50 °C (or room temperature). The 3:1 (v/v) ethanol-water-based electrolyte solution contained 1,3,5-benzene-tricarboxylic acid (H_3_BTC, 10.51 g l^−1^, 0.05 mol l^−1^) as precursor for the linker molecules and methyl-tributyl-ammonium methyl-sulphate (MTBS, 18.69 g l^−1^, 0.06 mol l^−1^).

Furthermore, copper foil was anodised under similar conditions (0.5 V *vs*. MSE, 10 min, 50 °C) in a 3:1 (v/v) ethanol-water-based electrolyte solution containing only the conducting additive MTBS (18.69 g l^−1^, 0.06 mol l^−1^) to obtain a porous and rough copper oxide/copper foil surface. Two self-made electrochemical Teflon cells were used for the two different electrolytes avoiding contamination and ensuring that a defined circular area (diameter: 10 mm) of a working electrode is exposed to the respective electrolyte. The setup was placed inside a drying oven with an internal thermometer to adjust the temperature and to represent a Faradaic cage. After electrochemical treatment, the foils were rinsed with deionised water and ethanol prior to drying in air with a blow-dryer.

Before infusion with the Krytox oil, the samples were vacuum-dried for 3 hours. The perfluorinated oil was then applied in a glove box until the samples were completely covered by the oil. After 10 min, the oil-covered samples were removed from the inert gas atmosphere. The excess oil was removed from the samples by blowing through nitrogen gas.

### Characterisation

XRD patterns of native, anodised and Cu-BTC-coated Cu foils were obtained in reflection mode with a Philips 1050 Diffractometer using Co K_α_ radiation (λ = 1.790307 Å). The measurement range 5° ≤ 2*θ* ≤ 50° allows to investigate copper oxide and MOF phases on the foil surface while avoiding the main reflections of copper. Structure analysis for the Cu-BTC coatings were performed with Fullprof implemented in WinPlotR according to the Rietveld method using the structure model of HKUST-1^36^ (lattice parameter a = 26.3357(11) Å, V = 18.266(2) Å^3^, space group *Fm*-3*m*). VESTA^68^ was used to prepare the drawing of the HKUST-1 crystal structure in Fig. 1.

The 3D-topography was characterised by a MicroProf optical profilometer from Fries Research Technology (FRT, Bergisch-Gladbach, Germany). The roughness was evaluated using the software package FRT Mark III V3.9.15 from the same company. Measurements were conducted at three positions on a Cu-BTC film prior to oil infusion and one area was measured on the Cu substrate foil for comparison. The scan length was defined to 1 mm · 1 mm and 100 · 100 points at a sampling rate of 100 Hz for the Cu-BTC film and 300 Hz for the Cu foil, respectively. One additional topography was measured at higher lateral resolution (100 points at 0.2 mm) to prove the independence of the evaluated roughness parameters of the measurement conditions. Roughness values *R*_*a*_ (arithmetical average roughness), *R*_*q*_ (root mean squared roughness), *R*_*p*_ (maximum peak height) and *R*_*v*_ (maximum valley depth) were calculated from the 3D-plot after leveling. 3D-picture evaluation was preferred over a 2D one with respect to the higher reliability of the values by avoiding errors induced by the manual line-selection of the evaluation region.

Raman spectra of native and anodised Cu foils were measured with a Thermo Scientific DXR Smart Raman spectrometer using an excitation wavelength of 532 nm and a spot size of 2.1 μm.

The morphologies of film and foil surfaces were investigated by scanning electron microscopy prior to oil-infusion using a Gemini Leo 1530 from Zeiss with field-emission gun. The specimens were characterised as obtained after the electrochemical surface modification without any pre-treatment such as applying a thin conductive coating. Due to the low electrical conductivity of MOF films, charging and edge effects make imaging more challenging, especially considering the fine surface structure at high magnifications. Thus, detailed information about the applied settings are provided in the supplementary information for each shown SE micrograph (Supplementary Table 5).

Environmental scanning electron microscopy was performed in a Quanta FEG 250 by Thermo Fisher Scientific (formerly FEI) equipped with a Peltier cooler. Infused samples were characterised after 30 min stability testing by water spraying (settings in Supplementary Table 6).

Contact angles were determined using a Krüss model DSA100 drop shape analyser for droplet dosing and image capturing, and the Krüss Advance software for profile detection and drop shape analysis. All contact angles were measured in the presence of air at temperatures ranging from 20 °C to 25 °C. Before a drop of a test fluid (water, diiodomethane or acetone) was placed, it was ensured that drops from previous measurements evaporated completely. The static contact angles were measured within 2 seconds after the placement of the drop. All obtained values are averaged over at least 15 measurements on at least 3 individual drops of the corresponding test fluid. Dynamic contact angles (i.e. advancing and receding contact angles) were measured by increasing and reducing the volume of a sessile drop at a flow rate of 2.7 μl s^−1^.

Spray tests were performed to assess the stability of the oil trapping. A nozzle was used to disperse water into a fine spray. The nozzle was placed at a distance of 15 cm in front of the vertically oriented samples and the water was sprayed directly at the samples. To ensure similar conditions for the spray test, all samples were placed next to each other and tested in a single test run.

## Electronic supplementary material

Supplementary Information

## Data Availability

The datasets generated during and/or analysed during the current study are available from the corresponding author on reasonable request.
