# Supplementary material for: Electrodeposited metal-organic framework films as self-assembled hierarchically superstructured supports for stable omniphobic surface coatings
Source: Sci Rep. 2018 Oct 18;8:15400. doi: 10.1038/s41598-018-33542-4 (PMC6194076; doi:10.1038/s41598-018-33542-4)
Supplement: Supplementary file 1 — Supplementary Information [file 41598_2018_33542_MOESM1_ESM.pdf]

Supplementary Information

Electrodeposited metal-organic framework films as self-assembled hierarchically superstructured supports for stable omniphobic surface coatings

Jakob Sablowski[1+], Julia Linnemann[2,3*+], Simone Hempel[4], Volker Hoffmann[2], Simon Unz[1], Michael Beckmann[1], and Lars Giebeler[2]

Optical Profilometry

Optical profilometry measurements were conducted to determine the roughness of electrodeposited Cu-BTC films in comparison to a native copper foil.

Supplementary Table 1 | Optical profilometry of an electrodeposited MOF film. Surface roughness parameters R_a (arithmetical average roughness), R_q (root mean squared roughness), R_p (maximum peak height), and R_V (maximum valley depth) for native Cu foil and electrodeposited Cu-BTC films.

Sample	Scan length (mm)	R_a (µm)	R_q (µm)	R_p (µm)	R_v (µm)
Cu foil	1.0	0.24	0.47	10.68	4.81
Cu-BTC film	1.0	1.92	2.43	10.64	8.50
Cu-BTC film	1.0	2.30	2.86	12.08	10.02
Cu-BTC film	0.2	2.48	3.05	10.57	8.59
Cu-BTC film average		2.23 ± 0.24	2.78 ± 0.26	11.1 ± 0.7	9.04 ± 0.70

[1] Chair of Energy Process Engineering, Institute of Process Engineering and Environmental Technology, Technische Universität Dresden, 01062 Dresden, Germany.
[2] Leibniz Institute for Solid State and Materials Research (IFW) Dresden e.V., Institute for Complex Materials, Helmholtzstraße 20, 01069 Dresden, Germany.
[3] Chair of Physical Chemistry, Technische Universität Dresden, Bergstraße 66b, 01069 Dresden, Germany.
[4] Institute of Construction Materials, Technische Universität Dresden, Georg-Schumann-Straße 7, 01187 Dresden, Germany.
* j.linnemann@ifw-dresden.de, julia.linnemann@e-chemistry.de
+ These authors contributed equally to this work

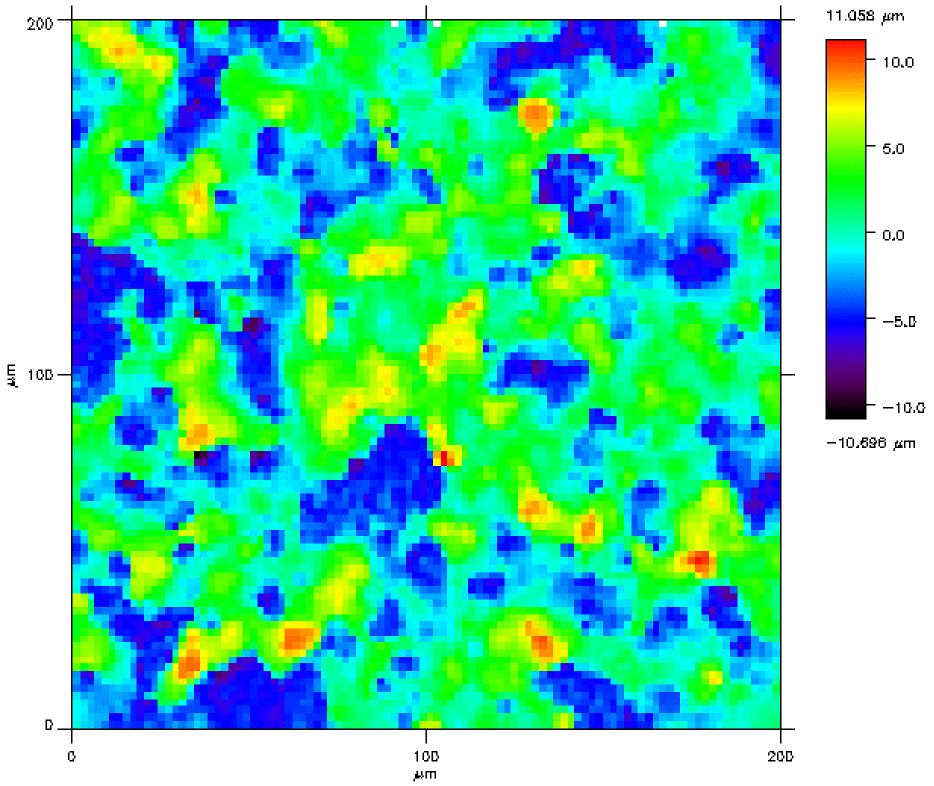

Supplementary Figure 1 | Optical profilometry image of an electrodeposited MOF film.

Additional scanning electron microscopy (SEM) image

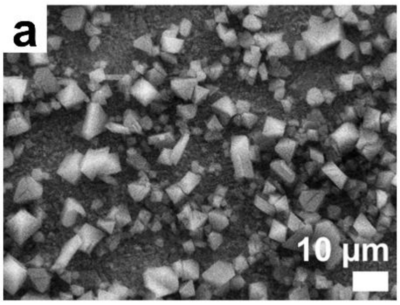

Supplementary Figure 2 | Morphology of a MOF film electrodeposited at room temperature. (**a**) SEM image of a Cu-BTC framework film electrodeposited at room temperature showing the top view. The scale bar is 10 µm.

Raman spectroscopy

The Raman spectrum of anodised copper foil (Supplementary Fig. 3a) contains the characteristic bands for cuprous oxide (Cu_2O) which are assigned in Supplementary Table 2. The features at 413 cm^{-1} and 710 cm^{-1} have been observed for Cu_2O films before[1].

The weak vibration signal around 305 cm^{-1} indicates the presence of cupric oxide (CuO). The CuO signal around 640 cm^{-1}, which was also observed for the native Cu foil (Supplementary Fig. 3b, Table 3), overlaps with the Cu_2O signal at ca. 630 cm^{-1}. The Raman active mode of CuO at 346 – 350 cm^{-1} reported in the literature[2, 3] does neither appear for the Cu foil nor the anodised Cu foil.

The Raman measurements further confirm the results of the XRD investigations. Mainly Cu_2O forms during the anodisation of Cu foil in an electrolyte only containing solvent and the additive methyl-tributyl-ammonium methyl-sulphate (MTBS).

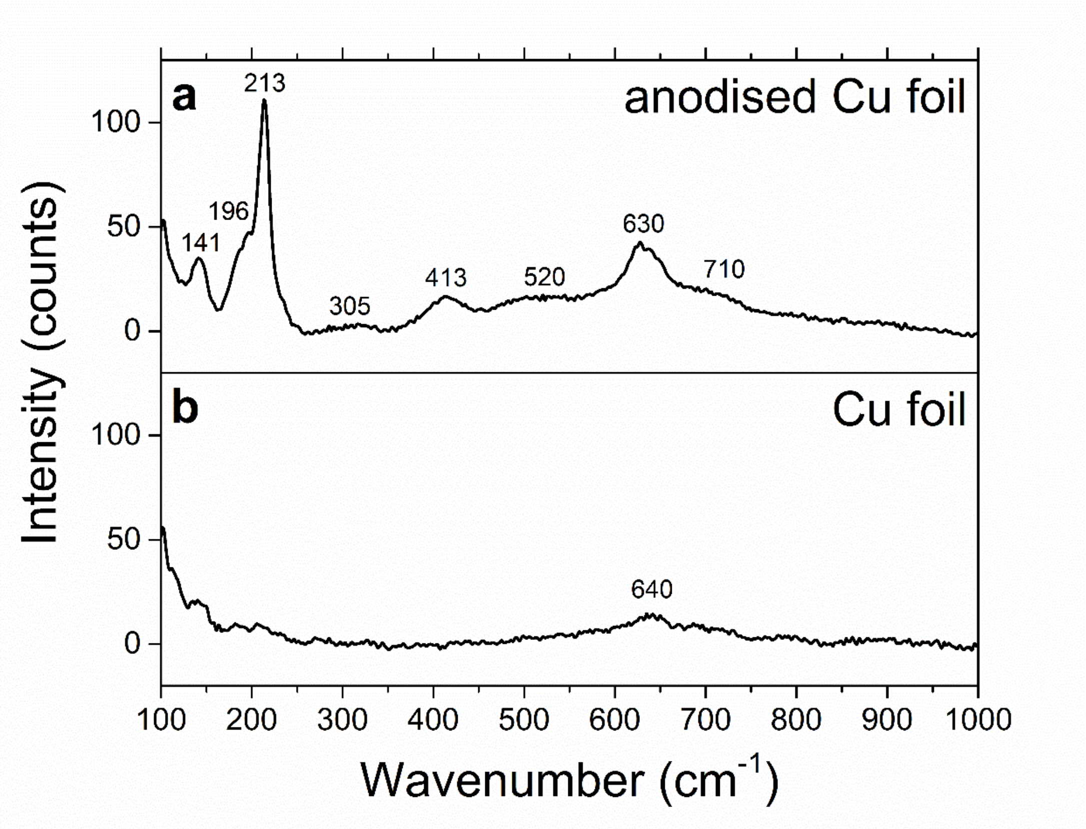

Supplementary Figure 3 | Raman spectra of Cu foils. (**a**) a Cu foil surface anodised in an electrolyte containing MTBS as conducting salt in EtOH/H_2O (3:1) and (**b**) a native Cu foil.

Supplementary Table 2 | Band assignment for cuprous oxide (Cu_2O). Bands observed in the Raman spectrum of anodised Cu foil (Supplementary Fig. 3a) are assigned to zone-center (Γ) phonon modes in Cu_2O (space group *Pn*-3*m*, 2 molecules in the unit cell) according to references[3, 4].

Band (cm^{-1})	Assignment
141	Out-of-phase beating of two Cu pairs
213	Rotation of the Cu tetrahedron about its centre with the upper two atoms moving out of phase with the lower two
520	Shearing of O planes (T_{2g} symmetry)
630	Beating of the Cu and O sublattices

Supplementary Table 3 | Band assignment for cupric oxide (tenorite, CuO). Bands observed in the Raman spectra of Cu foil and anodised Cu foil (Supplementary Fig. 3) are assigned to zone-center (Γ) phonon modes in CuO (space group *C*2/*c*, 4 molecules in the unit cell) according to references[2, 3].

Band (cm^{-1})	Assignment
305	Motion of oxygen atoms with displacements in the *b*-direction (A_g symmetry)
640	Motion of oxygen atoms with displacements perpendicular to the *b*-axis (B_g symmetry)

Droplet Sinking on oil-infused samples

Visible sinking of a droplet is observed after ca. 30 seconds on the oil-infused surfaces.

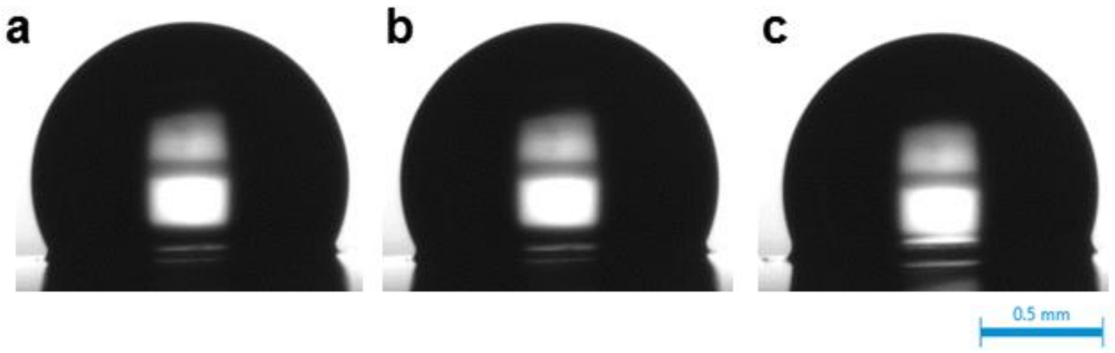

Supplementary Figure 4 | Droplet sinking on an oil-infused MOF film. Water drop after (**a**) 3 seconds, (**b**) 4 seconds and (**c**) 30 seconds an oil-infused Cu-BTC framework film.

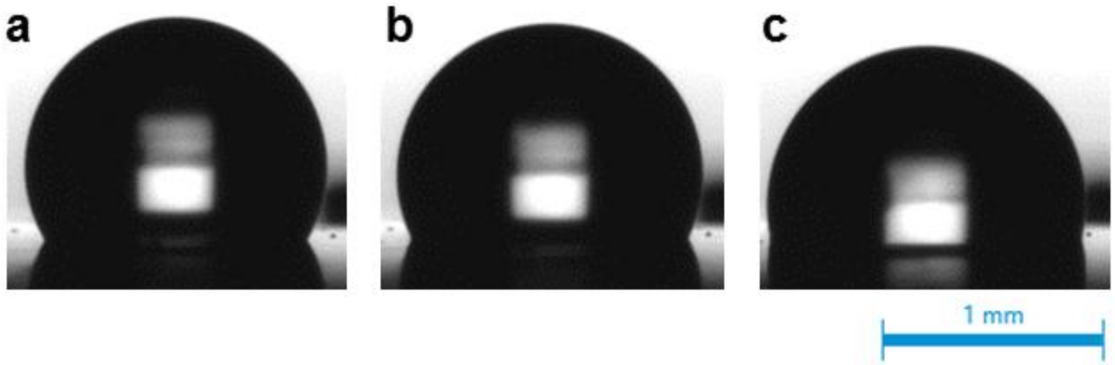

Supplementary Figure 5 | Droplet sinking on an oil-infused anodised Cu foil. Water drop after (**a**) 3 seconds, (**b**) 5 seconds and (**c**) 31 seconds on an oil-infused Cu foil anodised in an electrolyte not containing linker molecules**.**

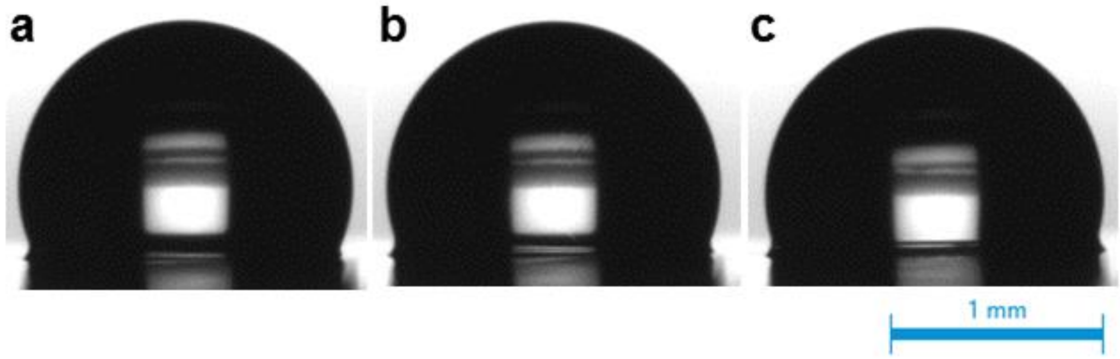

Supplementary Figure 6 | Droplet sinking on an oil-infused native Cu foil. Water drop after (**a**) 3 seconds, (**b**) 4 seconds and (**c**) 30 seconds on an oil-infused native Cu foil.

Supplementary detailed information on methods

Materials: Copper foil

The Copper foil produced in-house by rolling and annealing of electrolytic copper were subjected to elemental analysis, energy dispersive X-ray spectroscopy (EDXS) and X-ray diffraction (XRD) measurements to characterise the purity of the foil and the foil surface.

Elemental analysis

Two samples of the Cu foil (50 mg each) were dissolved in either 5 ml of a 1:1 (v/v) HNO_3/H_2O solution or 5 ml of a 5:5:1 (v/v/v) $HNO_3/H_2O/HF$ solution to determine the content of possible contamination elements by inductively coupled plasma optical emission spectrometry (ICP-OES) using a Thermo Scientifc IRIS Intrepid II XUV. The digestion solutions were filled up with water until 50 g were reached. None of the following elements was detected: Ag, Al, B, Be, Ca, Co, Cr, Fe, K, Li, Mg, Mn, Na, Ni, Pb and Zn (detection limit < 10 ppm mass fraction in the solid).

EDXS

According to the EDXS analysis, there may be a very low amount of iron contamination on the surface of the Cu foil (Supplementary Table 4 and Supplementary Fig. 7) which (if present) was assumingly introduced during the rolling process. The energy dispersive X-Ray spectrometer Bruker XFlash Detector 4010 was connected to a FEG Gemini Leo 1530 scanning electron microscope from Zeiss.

Supplementary Table 4 | EDXS characterisation results of the Cu foil. Measurements were performed at different parts of the Cu foil. The Software Quantax Esprit 2.1 was used for quantitative analysis of the spectra considering Cu and Fe.

Copper content (norm. mass%)	99.2	99.1	99.2	99.2	99.2
Iron content (norm. mass%)	0.8	0.9	0.8	0.8	0.8

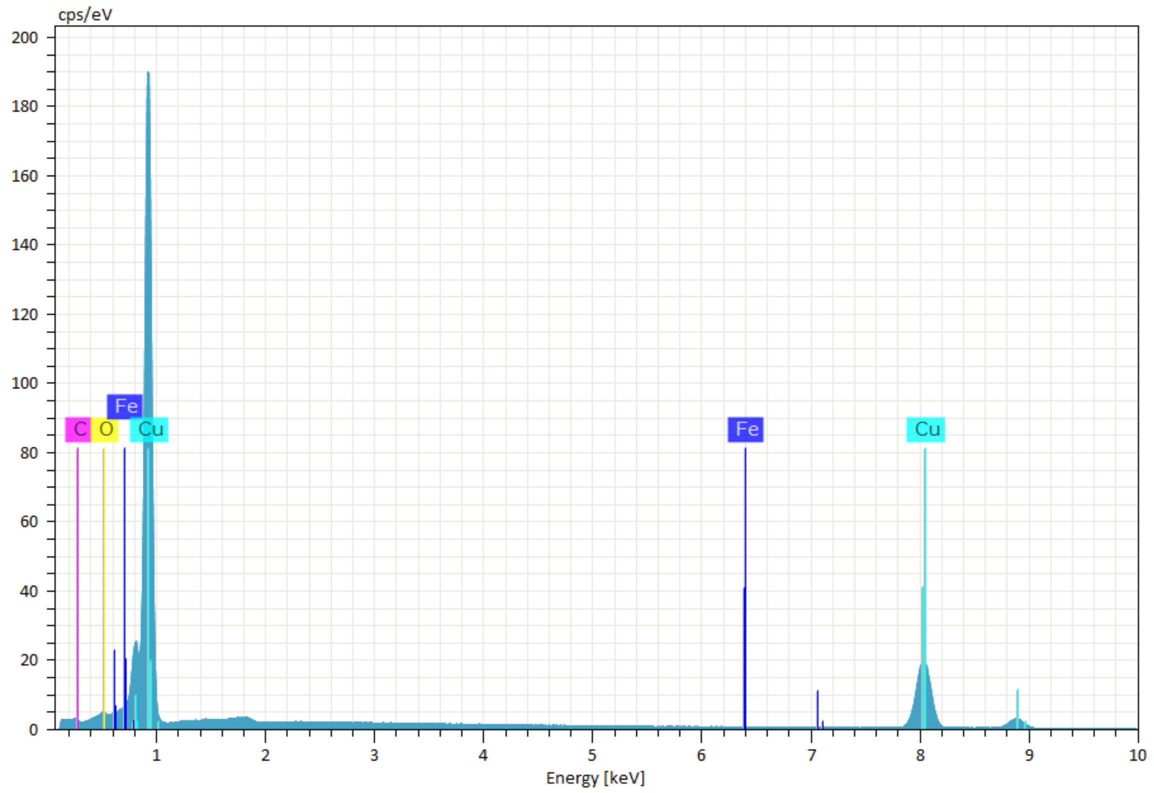

Supplementary Figure 7 | An EDX spectrum of the Cu foil.

XRD

XRD on a piece of copper foil in a wider 2θ range (5 ° ≤ 2θ ≤ 88 °) reveals signals assigned to Cu_2O *Pn*-3*m* and CuO *C*2/*c* (Supplementary Fig. 8). The signals at 48.3 ° 2θ and 53.4 ° 2θ may be due to contaminations on the surface caused by the rolling process. The measurements were conducted in reflection mode with a Philips 1050 Diffractometer using Co K_α radiation (λ = 1.790307 Å). The 2θ ranges of 50.5 ° ≤ 2θ ≤ 52 ° and 59 ° ≤ 2θ ≤ 60.5 ° were omitted during the XRD measurement as the signals of copper (bulk of the foil) appear in these ranges with very high intensities under the measurement conditions chosen to be able to observe signals of the foil surface (step size: 0.0130 ° 2θ, counting time: 248.37 s, aperture opening: 10 mm).

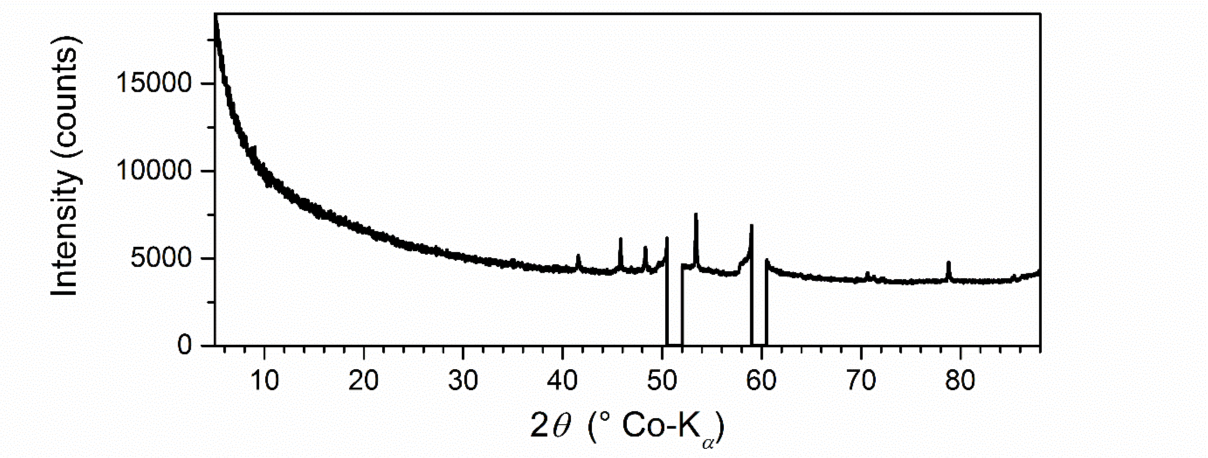

Supplementary Figure 8 | XRD pattern of the Cu foil.

Characterisation

SEM

A 30 µm - aperture was used. The high current mode of the condenser lens was deactivated minimising the spot size of the beam. Some images were recorded at intermediate scan rate with frame averaging.

Supplementary Table 5 | Settings used for SEM measurements.

Figure	Magnification	Accelerating Voltage (kV)	Detection	Working distance (mm)
2a	500 x	20	Secondary electrons (SE)	3.0
2b	5,000 x	5	SE - Inlens	3.0
2c	60,000 x	5	SE - Inlens	3.0
2d	20,000 x	15	SE - Inlens	3.2
3d	1,000 x	20	SE	3.0
3e	1,000 x	15	SE	6.0
3f	1,000 x	15	SE	3.2

ESEM

Supplementary Table 6 | Settings used for ESEM measurements.

Figure	5a	5b
Magnification	1,000 x	10,000 x
Accelerating Voltage (kV)	11.2	10.0
Spot size	2.5	4.0
Detection	SE	SE
Working distance (mm)	6.8 mm	6.8 mm
Humidity	20 %	60 %
Pressure (Pa)	131	455
Temperature	1 °C	3 °C

References

1. Fallberg A, Ottosson M, Carlsson J-O. Phase stability and oxygen doping in the Cu–N–O system. *J Cryst Growth* **312**, 1779-1784 (2010).

2. Chrzanowski J, Irwin JC. Raman scattering from cupric oxide. *Solid State Commun* **70**, 11-14 (1989).

3. Debbichi L, Marco de Lucas MC, Pierson JF, Krüger P. Vibrational Properties of CuO and Cu_4O_3 from First-Principles Calculations, and Raman and Infrared Spectroscopy. *J Phys Chem C* **116**, 10232-10237 (2012).

4. Reimann K, Syassen K. Raman scattering and photoluminescence in Cu_2O under hydrostatic pressure. *Phys Rev B* **39**, 11113-11119 (1989).
